# Comparative Evaluation of β-TCP-Based Composite Biomaterials Using Chorionic Mesenchymal Stem Cells Under Non-Osteogenic Conditions

**DOI:** 10.3390/polym18121543

**Published:** 2026-06-21

**Authors:** Jana Čajková, Marianna Trebuňová, Darina Bačenková, Gabriela Ižaríková, Erik Dosedla, Jozef Živčák

**Affiliations:** 1 Department of Biomedical Engineering and Measurement, Faculty of Mechanical Engineering, Technical University of Košice, 04200 Košice, Slovakia; marianna.trebunova@tuke.sk (M.T.); darina.bacenkova@tuke.sk (D.B.); jozef.zivcak@tuke.sk (J.Ž.); 2 Department of Applied Mathematics and Informatics, Faculty of Mechanical Engineering, Technical University of Košice, Letná 9, 04200 Košice, Slovakia; gabriela.izarikova@tuke.sk; 3 Department of Gynecology and Obstetrics, Faculty of Medicine, Pavol Jozef Šafarik University Hospital, AGEL Košice-Šaca, Pavol Jozef Šafarik University in Košice, 04015 Košice-Šaca, Slovakia; erik.dosedla@nke.agel.sk

**Keywords:** chorion-derived mesenchymal stem cells, beta-tricalcium phosphate, bone tissue engineering, biodegradable polymers, poly(lactide-co-caprolactone), poly(lactic-co-glycolic), zinc oxide

## Abstract

This study evaluates the intrinsic osteogenic potential of β-tricalcium phosphate (β-TCP)-containing composite scaffolds (PLCL–TCP, PLGA–TCP, and ZnO–TCP) on chorion-derived mesenchymal stem cells (CMSCs) under non-osteogenic in vitro conditions. CMSCs were cultured on the three biomaterials for 35 days without osteogenic supplements to isolate the material-driven cellular response. Cell viability was assessed via MTT assay, while osteogenesis-associated markers (alkaline phosphatase, type I collagen, and osteocalcin) were quantified using ELISA. Scaffold surface morphology and elemental composition were characterized before and after cultivation utilizing SEM and EDX. All investigated scaffolds supported long-term CMSC viability and induced measurable osteogenic responses. PLCL–TCP demonstrated a consistently strong biological response, characterized by sustained metabolic activity, elevated ALP and COL I production, and increased osteocalcin levels at later stages of cultivation. ZnO–TCP also exhibited favorable osteogenesis-associated responses, particularly with respect to late-stage osteocalcin production, while maintaining high structural stability. In conclusion, β-TCP composites can intrinsically modulate CMSC behavior without biochemical supplements. Osteogenic outcomes depend on a complex interplay of surface chemistry, scaffold architecture, and degradation profiles, with PLCL–TCP demonstrating favorable overall performance among the investigated biomaterials.

## 1. Introduction

Among the biomaterials investigated for bone tissue engineering, calcium phosphate-based ceramics have attracted considerable attention because of their chemical similarity to the mineral phase of bone [1,2]. In particular, β-tricalcium phosphate (β-TCP) is widely recognized for its osteoconductive properties, biocompatibility, and biodegradability [3]. Numerous studies have demonstrated that β-TCP-containing materials support cell attachment, proliferation, and osteogenic differentiation while serving as a source of calcium and phosphate ions that participate in bone remodeling processes [4]. In addition to their osteoconductive role, calcium phosphate surfaces can directly influence mesenchymal stem cell behavior through physicochemical interactions at the cell–material interface [5,6].

Early studies established the ability of calcium phosphate ceramics to support bone formation both in vitro and in vivo. Yuan et al. demonstrated that porous calcium phosphate ceramics promoted ectopic bone formation and supported osteogenic differentiation, highlighting the importance of scaffold architecture and chemistry in regulating cellular responses [7,8]. Similarly, LeGeros et al. emphasized the biological significance of calcium phosphate biomaterials and their capacity to mimic the mineral phase of bone, thereby facilitating tissue integration and regeneration [9].

Increasing evidence indicates that calcium phosphate biomaterials can provide intrinsic osteogenic cues independent of soluble differentiation factors. Müller et al. demonstrated that calcium phosphate substrates stimulated osteogenic marker expression even in the absence of osteogenic supplements [10]. Similarly, Shih et al. reported that calcium phosphate-containing matrices can regulate stem cell fate through material-driven signaling pathways [11]. These findings support the concept that scaffold composition itself may direct osteogenic differentiation without the addition of exogenous biochemical factors. The importance of calcium phosphate surface characteristics has been further emphasized by Xiao et al., who highlighted that osteogenic performance is strongly influenced by crystal morphology, roughness, porosity, surface energy, and ion exchange behavior [12]. These physicochemical properties affect protein adsorption, focal adhesion formation, cytoskeletal organization, and intracellular signaling pathways involved in osteogenesis.

The importance of these structural parameters has been extensively discussed by Turnbull et al., who emphasized that pore architecture, interconnectivity, scaffold geometry, and surface morphology strongly influence cellular attachment, proliferation, differentiation, and tissue ingrowth [13]. Together, these findings indicate that successful osteoinduction depends not only on the chemical composition of calcium phosphate phases but also on their ability to create a favorable microenvironment for stem cell attachment and differentiation [14].

To overcome the brittleness and limited mechanical performance of calcium phosphate ceramics, increasing attention has been directed toward composite scaffolds that combine calcium phosphates with biodegradable polymers [15]. Such composites aim to integrate the bioactivity of calcium phosphate phases with the flexibility, processability, and tunable degradation profiles of polymeric matrices [16]. Recent reviews, including Baino et al., have highlighted the advantages of combining biodegradable polymers with calcium phosphate ceramics to improve both biological and mechanical performance. Among the polymers investigated, poly(L-lactide-co-caprolactone) (PLCL) and poly(lactic-co-glycolic acid) (PLGA) have demonstrated considerable potential for bone tissue engineering applications [17]. Farahani et al. summarized the ability of PLGA-based systems to support osteogenesis and controlled degradation [18]. Recent studies have reported that PLGA/TCP composites enhance cell attachment, proliferation, and mineralization while providing improved mechanical stability compared with ceramic-only scaffolds [18]. Likewise, PLCL-based composites have attracted interest because their elasticity and degradation behavior more closely resemble those of native tissues, supporting favorable cellular responses and tissue integration [19,20,21,22].

In addition to polymer–ceramic composites, the incorporation of bioactive trace elements has emerged as an effective strategy for enhancing scaffold performance. Zinc is an essential micronutrient involved in bone metabolism and plays important roles in osteoblast proliferation, differentiation, and mineralization [23,24]. The biological functions of zinc and its beneficial effects on bone regeneration were comprehensively reviewed by Su et al., where they reported that zinc incorporation into calcium phosphate-based materials can enhance osteogenic differentiation, mineral deposition, and new bone formation [22].

Alongside scaffold design, the selection of an appropriate stem cell source is a critical factor in bone tissue engineering [23]. Although bone marrow-derived mesenchymal stem cells (BM-MSCs) remain the most extensively studied cell type for bone regeneration [24], their clinical application is limited by invasive harvesting procedures, donor variability, and age-related declines in regenerative capacity [25]. Perinatal tissues represent a particularly attractive source of mesenchymal stem cells because they are readily available, ethically acceptable, and obtained without invasive procedures [26]. Mihu et al. highlighted the high proliferative capacity, multilineage differentiation potential, immunomodulatory properties, and low immunogenicity of placenta-derived MSC populations [27]. Chorion-derived mesenchymal stem cells (CMSCs) are particularly attractive because they combine these advantages with ease of isolation and minimal ethical concerns [28].

The present study investigated the biological response of CMSCs cultured on three β-TCP-containing scaffolds (PLCL–TCP, PLGA–TCP, and ZnO–TCP) under non-osteogenic conditions. These materials were selected as representative β-TCP-based biomaterials developed for bone tissue engineering applications, differing in matrix composition, structural organization, and surface characteristics while sharing the common presence of the bioactive β-TCP phase. Cell viability was assessed using the MTT assay, while osteogenesis-associated responses were evaluated through quantification of alkaline phosphatase (ALP), type I collagen (COL I), and osteocalcin (OC). In addition, scaffold surface morphology and elemental composition before and after cell cultivation were characterized using SEM and EDX analyses. By excluding osteogenic supplements from the culture system, this study aimed to evaluate the intrinsic ability of β-TCP-containing scaffolds to influence CMSC behavior and osteogenesis-associated responses, while correlating biological outcomes with scaffold surface characteristics. While PLCL, PLGA, and ZnO have been previously studied, the main highlight of this study is its evaluation the intrinsic osteogenic potential of the materials themselves. By directly comparing these β-TCP-based composite scaffolds from multiple analytical perspectives—combining biological assessments of cell viability (MTT) and osteogenic marker secretion (ELISA) with structural and elemental characterization (SEM, EDX)—this approach contributes to a better understanding of the interactions between chorionic stem cells, utilized as an in vitro model, and biomaterials. Ultimately, it provides an insight into how specific scaffold compositions and surface properties drive CMSC osteogenic behavior without external osteogenic stimulation.

## 2. Materials and Methods

### 2.1. Isolation of Chorionic Mesenchymal Stem Cells (CMSCs)

The cellular model used in this study was based on chorion-derived mesenchymal stem cells that had been previously isolated, characterized, and evaluated by our group (Bačenková et al., 2011; 2020) [29,30]. The objective of the present work was to compare the biological performance of the investigated biomaterials using a well-defined and previously validated cell source. The collection and use of human placental chorion tissue were performed in accordance with the Declaration of Helsinki and approved by the Ethics Committee of the AGEL Košice-Šaca Hospital and the Faculty of Medicine, Pavol Jozef Šafárik University in Košice (Approval No. 11-2021). Written informed consent was obtained from a donor prior to tissue collection. All experiments were performed using cells derived from a single placental isolation batch to ensure the consistency of the cellular population and minimize potential donor-related variability. The experimental replicates for all subsequent assays (MTT and ELISA) were technical replicates derived from this primary cell bank, ensuring that observed differences in cellular response were attributable solely to the material composition. Isolation and in vitro cultivation of CMSCs were performed using placental tissues obtained after planned Cesarean section delivery. Fetal membranes were collected under sterile conditions in the gynecological operating room immediately after delivery and stored in cooled collection medium. The collection medium consisted of DMEM (Thermo Fisher Scientific, Gibco, Carlsbad, CA, USA) supplemented with 2% fetal bovine serum (Thermo Fisher Scientific, Gibco, California, Carlsbad, CA, USA) and 1% antibiotic-antimycotic solution (Thermo Fisher Scientific, Gibco, Carlsbad, CA, USA). After transport to the laboratory, the tissue was processed under strictly sterile conditions in a laminar flow cabinet (ESCO Sentinel Gold, Esco Micro Pte. Ltd., Singapore). The amnion was mechanically separated from the chorion using surgical forceps in a Petri dish containing culture medium. This step enables selective processing of chorionic tissue intended for subsequent enzymatic digestion and cell isolation. The chorionic tissue was subsequently fragmented into smaller pieces approximately 2 × 2 cm in size.

For enzymatic cell isolation, a two-step digestion protocol modified according to Soncini et al. was used [31]. The chorionic fragments were first incubated in a dispase solution with a concentration of 2.4 U/mL (Thermo Fisher Scientific, Gibco, Carlsbad, CA, USA) prepared in DMEM (Thermo Fisher Scientific, Gibco, Carlsbad, CA, USA) supplemented with antibiotics (antibiotic/antimycotic, Gibco, Carlsbad, CA, USA) at 37 °C for 10 min (ESCO CelCulture^®^ CO_2_, Singapore). Following incubation, centrifugation (LMC-4200R, BIOSAN, Riga, Latvia) was performed at 1300 rpm for 15 min at laboratory temperature, followed by removal of the supernatant. The tissue was subsequently washed in DMEM (Thermo Fisher Scientific, Gibco, Carlsbad, CA, USA), and centrifugation was repeated. After washing, a preheated collagenase type II solution at a concentration of 1 mg/mL was added to the fragments, and the samples were incubated at 37 °C for 90 to 120 min depending on the degree of tissue dissociation. Following enzymatic digestion, repeated washing in DMEM (Thermo Fisher Scientific, Gibco, Carlsbad, CA, USA) and centrifugation at 1300 rpm for 15 min at 4 °C were performed.

The resulting cell suspension containing released cells and residual tissue fragments was filtered through a cell strainer with a pore size of 40 μm. After filtration, the suspension was centrifuged, and the obtained cell sediment was considered the population of chorionic mesenchymal stem cells. The cell pellet was resuspended in a precisely defined volume of complete culture medium (Table 1) consisting of a 1:1 mixture of DMEM and α-MEM (Thermo Fisher Scientific, Gibco, Carlsbad, CA, USA), supplemented with 10% fetal bovine serum (Thermo Fisher Scientific, Gibco, Carlsbad, CA, USA) and 1% antibiotic-antimycotic solution (Thermo Fisher Scientific, Gibco, Carlsbad, CA, USA).

### 2.2. Flow Cytometry (FACS) of CMSCs

Following the isolation of CMSCs from fetal membranes, comprehensive characterization was performed to confirm the identity and purity of the obtained cell population. This step was carried out to ensure that the isolated cells exhibited the required parameters and represented the correct target type of mesenchymal stem cells. Visual analysis of the CMSC monolayer culture after the first passage demonstrated a high degree of homogeneity. The cells exhibited a uniform fibroblast-like appearance characterized as “spindle-shaped,” while no presence of non-adherent hematopoietic cells was observed in the culture environment. To precisely confirm the phenotype, flow cytometry analysis was performed. The applied gating strategy was designed to primarily define the population of viable cells based on their size (FSC) and granularity (SSC). Subsequent application of singlet gating to exclude doublets enabled the evaluation of fluorescence intensity at the level of individual cells.

### 2.3. Preparation of Biomaterial Samples

**Figure 1:**
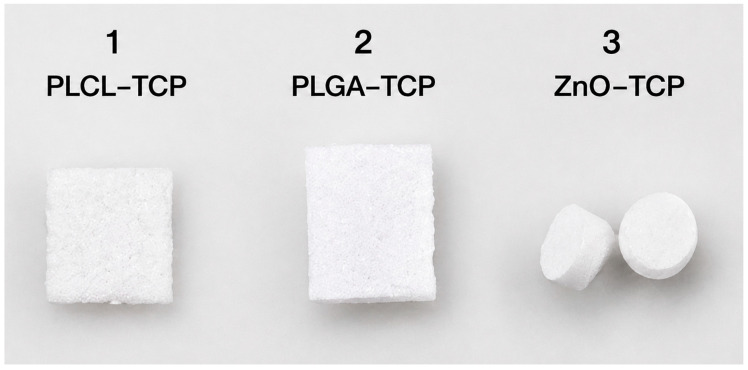
Commercially supplied biomaterial samples used in the study: (1) PLCL–TCP—poly(ε-caprolactone-co-lactide) with β-tricalcium phosphate, (2) PLGA–TCP—poly(lactide-co-glycolide) with β-tricalcium phosphate, and (3) ZnO–TCP—zinc oxide with β-tricalcium phosphate.

The biomaterial samples used in this study were commercially supplied by BONEGRAFT Biyolojik Malzemeler San. ve Tic. A.Ş. (Bornova/Izmir, Turkey) and provided in sterile, ready-to-use packaging. The selection included three types of osteoconductive composites: PLCL-TCP, consisting of a β-tricalcium phosphate -biodegradable polymer (PLC) matrix; PLGA-TCP, composed of a poly(lactide-co-glycolide) (PLGA) matrix combined with β-tricalcium phosphate; and ZnO-TCP, an inorganic composite consisting of β-tricalcium phosphate and zinc oxide (Figure 1). According to the manufacturer’s specifications, the β-tricalcium phosphate phase in these scaffolds exhibits a stoichiometric Ca/P ratio of 1.5, ensuring high chemical purity and phase stability. All materials were handled under strict aseptic conditions in a laminar flow cabinet (ESCO Sentinel Gold, Esco Micro Pte. Ltd., Singapore). Given their medical-grade sterile status confirmed by CE 1783 certification, no further sterilization was required. Individual samples were subsequently transferred into 24-well culture plates using sterile forceps, with each well containing one material disk to enable parallel comparison under identical culture conditions.

### 2.4. Seeding of Mesenchymal Stem Cells onto Biomaterial Samples

After reaching the desired 80% confluence, CMSCs were enzymatically detached from the culture vessel using trypsin-EDTA solution (Thermo Fisher Scientific, Gibco, Carlsbad, CA, USA) and subsequently centrifuged to remove residual culture medium. Following centrifugation, the cells were resuspended in culture medium consisting of DMEM (Thermo Fisher Scientific, Gibco, Carlsbad, CA, USA) and α-MEM (Thermo Fisher Scientific, Gibco, Carlsbad, CA, USA) in a 1:1 ratio, supplemented with 10% fetal bovine serum (Thermo Fisher Scientific, Gibco, Carlsbad, CA, USA) and 1% antibiotic-antimycotic solution (Thermo Fisher Scientific, Gibco, Carlsbad, CA, USA). Commercially purchased biomaterial samples were supplied as sterile solid materials and handled under aseptic conditions prior to the experiment. Cells were seeded at a density of 1 × 10^5^ cells/scaffold, which enabled efficient attachment and long-term cultivation throughout the experimental period. After seeding, the cells were incubated under standard culture conditions (37 °C, 5% CO_2_, saturated humidity ≥ 95%) in an incubator (Esco CelCulture^®^ CO_2_ Incubator, Esco Micro Pte. Ltd., Singapore). Cultivation of cells on the biomaterial samples was carried out for 35 days. The culture medium was replaced every 3 days under strictly sterile conditions to maintain a contamination-free environment.

### 2.5. Viability Analysis—MTT Assay

The metabolic activity of cells cultured on the tested biomaterials was evaluated using a colorimetric MTT assay (Thermo Fisher Scientific, Gibco, Carlsbad, CA, USA). This method is based on the reduction of the yellow tetrazolium substrate MTT (3-(4,5-dimethylthiazol-2-yl)-2,5-diphenyltetrazolium bromide) by mitochondrial dehydrogenases of metabolically active cells, resulting in the formation of insoluble formazan crystals [32]. The resulting formazan crystals were subsequently dissolved using dimethyl sulfoxide (DMSO, Sigma-Aldrich, St. Louis, MO, USA). The amount of formed formazan directly correlates with the metabolic activity of the cells and therefore indirectly reflects their viability and proliferation. The MTT assay was performed on days 3, 9, 14, 20, 27, and 35 of cultivation in order to monitor the dynamics of cellular metabolic activity during long-term interaction with the individual biomaterials. At each time point, the culture medium was removed from the wells, and the samples were gently washed with DMEM medium (Thermo Fisher Scientific) to remove residual serum and non-adherent cells. An MTT solution (typically at a concentration of 5 mg/mL in PBS) was added to each well in a volume corresponding to 10% of the culture medium volume. The samples were incubated in a CO_2_ incubator (Esco CelCulture^®^ CO_2_ Incubator, Esco Micro Pte. Ltd., Singapore) at 37 °C for approximately 3 h, allowing intracellular reduction of MTT into formazan crystals. After incubation, the MTT solution was removed, and the formed formazan crystals were dissolved using DMSO. Formazan solubilization was carried out overnight to ensure complete dissolution of the crystals and homogenization of the solution. Subsequently, the absorbance of the resulting solution was measured using a spectrophotometer at a wavelength of 490 nm. A blank sample consisted of a solution containing all reagents except cells, i.e., medium with MTT followed by DMSO, and was used for background absorbance correction. The control group consisted of cells seeded at the same time and from the same passage as the experimental samples but cultured directly on the surface of the culture well without the presence of biomaterial. This control represented the reference value of cellular metabolic activity under standard culture conditions. The measured absorbance values were corrected for the blank value and subsequently used for comparison of cellular metabolic activity on the individual tested materials depending on cultivation time. Differences in cell viability among the experimental groups and culture periods were analyzed using two-way ANOVA followed by Tukey’s multiple comparison test. Statistical significance was set at *p* < 0.05.

### 2.6. Quantitative Analysis of Osteogenic Markers (ELISA)

To evaluate the potential osteogenic response of CMSCs cultured on the investigated scaffolds, the secretion of alkaline phosphatase (ALP), type I collagen (COL I), and osteocalcin (OC) was quantified. These markers were selected to characterize different stages of osteogenesis-associated cellular activity (ALP), extracellular matrix formation (COL I), and late osteoblastic maturation (OC). In order to assess the ability of CMSCs to undergo spontaneous differentiation induced solely by the physicochemical properties of the biomaterial, cells were maintained throughout the entire 35-day experimental period in basal culture medium (Table 1) without supplementation with osteogenic differentiation factors. Culture supernatants were collected on days 14, 21, and 35, centrifuged at 1500 rpm for 10 min to remove cellular debris, aliquoted, and stored at −80 °C until analysis. As the cells were maintained under non-osteogenic culture conditions without supplementation with osteogenic differentiation factors, relatively low concentrations of osteogenesis-associated markers were expected. Therefore, culture supernatants were analyzed without prior dilution to maximize assay sensitivity and ensure reliable detection of the target analytes. Quantitative determination of ALP, COL I, and OC concentrations was performed using human-specific enzyme-linked immunosorbent assay (ELISA) kits (Human ALP ELISA Kit, EKU02240; Human Osteocalcin/OC ELISA Kit; and Human Collagen Type I ELISA Kit, all from Biomatik, Wilmington, DE, USA). These kits were selected for their high specificity and sensitivity in detecting osteogenesis-associated proteins in culture supernatants, as per the manufacturer’s instructions. Standard curves for each marker were generated using the provided standards, and all measurements were performed in triplicate to ensure analytical precision. Following incubation and washing steps, enzyme-conjugated detection antibodies were applied. Subsequently, tetramethylbenzidine (TMB) substrate solution was added, and color development was allowed to proceed according to the manufacturer’s protocol. The reaction was terminated using stop solution, and absorbance was measured at 490 nm using a microplate reader. Concentrations of individual analytes were calculated from standard calibration curves generated from serial dilutions of the supplied standards and expressed in the units specified by the respective assay kits. The concentrations of ALP, COL I, and OC were expressed as mean ± standard deviation (SD). Differences among experimental groups were analyzed using two-way analysis of variance (ANOVA), with biomaterial type and cultivation time as independent variables. The interaction between these factors was also evaluated. Pairwise comparisons were subsequently performed using Scheffé’s post hoc test. Statistical significance was defined as *p* < 0.05.

### 2.7. SEM Analysis of Sample Surface Morphology

Scanning electron microscopy (SEM) (TESCAN, Brno, Czech Republic) was used for the analysis of biomaterial surface morphology, enabling detailed evaluation of material topography and microstructure before and after the cultivation. Prior to measurement, the samples were fixed onto aluminum holders (stubs) using double-sided conductive carbon tape, ensuring both sample stability and electrical contact with the holder. Since the analyzed materials exhibited non-conductive properties, they were subsequently coated with a thin conductive carbon layer using carbon coating. This step was necessary to eliminate surface charging during measurement and to obtain high-quality imaging. SEM analysis was performed under high vacuum conditions, with image formation resulting from the interaction between a focused electron beam and the sample surface. Detection of secondary electrons provided information regarding surface morphology and topography, enabling evaluation of the structural characteristics of the materials and their changes following in vitro cell cultivation. It should be noted that imaging under high-vacuum conditions without specialized biological fixation or critical point drying protocols limits the preservation of delicate cellular structures and extracellular matrix (ECM). Consequently, the resulting micrographs primarily reflect modifications to the underlying scaffold architecture, surface remodeling, and inorganic deposits rather than intact biological coverage.

### 2.8. Morphological and Chemical Characterization (SEM and EDX)

EDX analysis coupled with SEM was used to evaluate the surface chemical composition of the investigated biomaterials. This method enables both qualitative and semi-quantitative analysis of elements present within the analyzed sample volume based on the detection of characteristic X-ray radiation emitted following the interaction of the primary electron beam with the material. EDX analysis was performed using area (mapping) spectra, and the obtained results were expressed in weight percentages (wt%). In relevant cases, conversion to atomic percentages (at%) was additionally used for the interpretation of stoichiometric ratios such as Ca/P. Since EDX analyzes the surface layer of the material, typically within the range of several micrometers depending on composition and voltage parameters, the obtained data represent the chemical composition of the exposed surface after experimental manipulation. The aim of the analysis was to confirm the presence of inorganic fillers such as β-TCP and ZnO, identify potential changes in surface chemistry following cell cultivation, and detect possible deposits or adsorbed components originating from the culture medium composed of α-MEM/DMEM supplemented with FBS and antibiotics. Given the highly porous and irregular three-dimensional topography of the investigated scaffolds, EDX was utilized as a semi-quantitative technique. Therefore, the reported weight percentages reflect relative elemental abundance and spatial distribution across the exposed surface layer rather than absolute bulk compositional values.

## 3. Results

### 3.1. Flow Cytometry (FACS) of CMSCs

**Figure 2:**
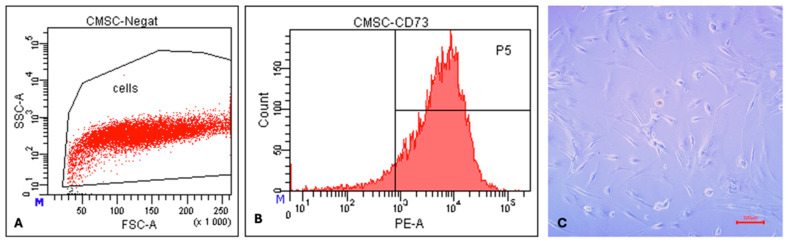
Characterization of chorion-derived mesenchymal stem cells (CMSCs). (**A**) Flow cytometric FSC/SSC dot plot showing the gating strategy used to identify the main CMSC population for subsequent analysis. (**B**) Representative flow cytometry histogram demonstrating high expression of the mesenchymal stem cell marker CD73. (**C**) Representative micrograph of CMSCs after the first passage exhibiting typical spindle-shaped fibroblast-like morphology and adherent growth characteristics. Scale bar = 100 μm.

Following isolation from human fetal membranes, CMSCs were subjected to phenotypic characterization to verify the identity, purity, and quality of the obtained cell population. Morphological evaluation after the first passage revealed a homogeneous adherent cell population exhibiting the typical spindle-shaped fibroblast-like morphology characteristic of mesenchymal stem cells (Figure 2C).

No significant presence of non-adherent hematopoietic cells was observed during cultivation. To confirm the cellular phenotype, flow cytometric analysis was performed using a sequential gating strategy. Initially, the main cell population was identified based on forward scatter (FSC-A) and side scatter (SSC-A) parameters, allowing exclusion of cellular debris, apoptotic bodies, and other impurities. Subsequently, a singlet gate was applied to eliminate cell aggregates and doublets, ensuring that only individual cells were included in the final analysis. Negative and isotype controls were used to determine the level of cellular autofluorescence and non-specific antibody binding. Based on these controls, the positivity threshold (P5 gate) was established for all analyzed markers. Both control samples exhibited low background fluorescence, allowing reliable discrimination between positive and negative cell populations. Immunophenotypic analysis confirmed a high expression of characteristic mesenchymal stem cell markers (Table 2).

The highest positivity was detected for CD73 (91.9%), followed by CD29 (88.3%) and CD44 (77.6%). In contrast, the combined expression of hematopoietic markers CD14, CD19, CD34, and CD45 was only 2.9%, indicating the absence of significant contamination by hematopoietic cells. The obtained immunophenotypic profile is consistent with the minimal criteria established by the International Society for Cell & Gene Therapy (ISCT) for mesenchymal stromal cells, which require the expression of characteristic mesenchymal markers together with minimal expression of hematopoietic markers. These findings therefore confirm the successful isolation of a highly enriched CMSC population with a typical mesenchymal phenotype.

### 3.2. Cell Viability Assessed by MTT Assay

**Figure 3:**
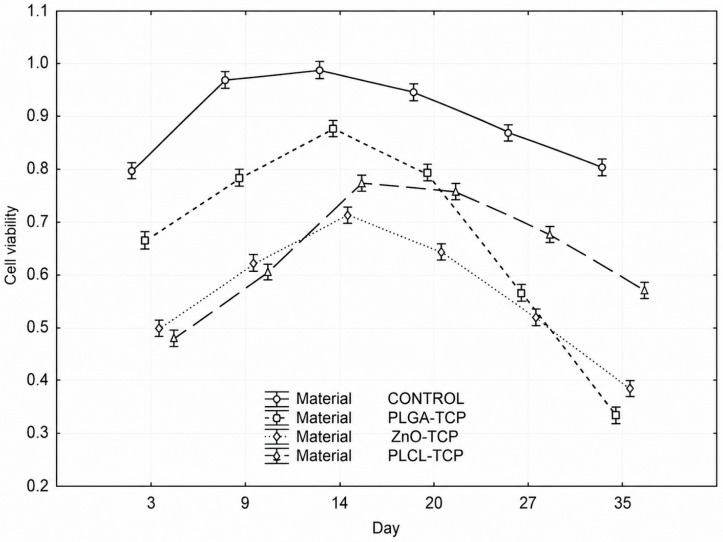
Cell viability of CMSCs cultured on PLCL–TCP, PLGA–TCP, and ZnO–TCP scaffolds during a 35-day cultivation period as determined by the MTT assay (absorbance at 490 nm). Data are presented as mean ± SD.

Cell viability of CMSCs cultured on β-TCP-containing composite biomaterials was evaluated using the MTT assay over a 35-day cultivation period (Figure 3). In all experimental groups, cell viability increased progressively from day 3 to day 14, indicating successful cell adhesion and proliferation, before subsequently declining. Maximum viability was reached at day 14 across all groups: Control (0.991), PLGA–TCP (0.877), PLCL–TCP (0.775), and ZnO–TCP (0.715).

The control group consistently exhibited the highest viability at all evaluated time points. Among the investigated biomaterials, PLGA–TCP showed the highest viability during the early cultivation period (days 3–20), reaching approximately 88.5% of the control value on day 14. However, a pronounced decrease was observed during prolonged cultivation. In contrast, PLCL–TCP maintained a comparatively more stable viability profile and exceeded both PLGA–TCP and ZnO–TCP during the later cultivation period (days 27–35). ZnO–TCP exhibited the lowest overall cell viability throughout the experiment.

Two-way ANOVA revealed significant effects of biomaterial type (*p* < 0.01) and cultivation time (*p* < 0.01) on cell viability, as well as a significant interaction between these factors (*p* < 0.01). This significant interaction indicates that the effect of the biomaterial on cell viability is not constant but changes depending on the cultivation time. Post hoc analysis confirmed significant differences among the majority of experimental groups, indicating that the temporal changes in cell viability were dependent on biomaterial composition.

### 3.3. Osteogenesis-Associated Marker Production

**Figure 4:**
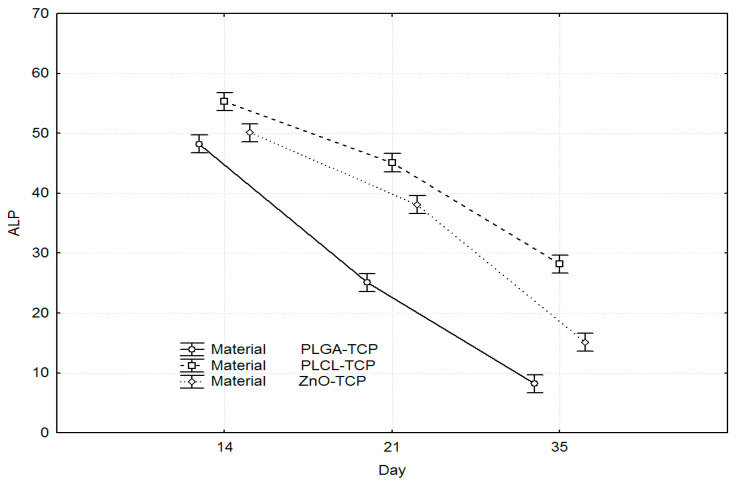
Osteogenesis-associated production of alkaline phosphatase (ALP) by CMSCs cultured on β-TCP-containing composite biomaterials under non-osteogenic conditions (concentration (ng/mL).

**Figure 5:**
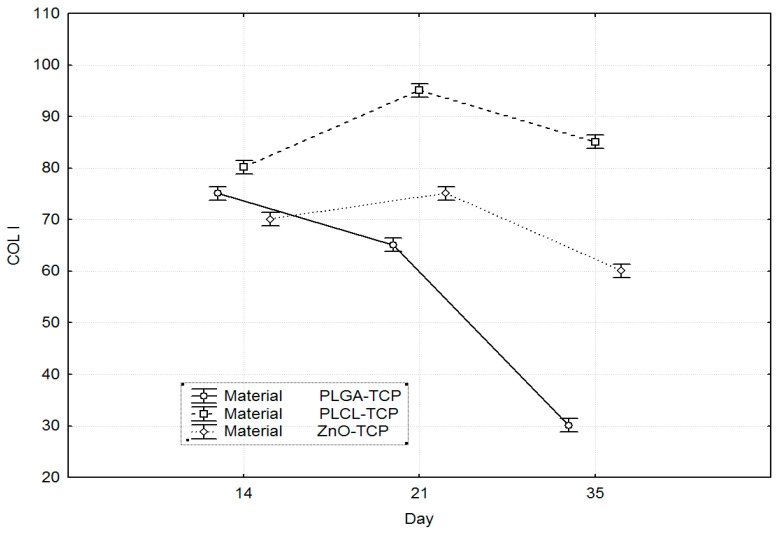
Osteogenesis-associated production of type I collagen (COL I) by CMSCs cultured on β-TCP-containing composite biomaterials under non-osteogenic conditions (concentration (ng/mL).

**Figure 6:**
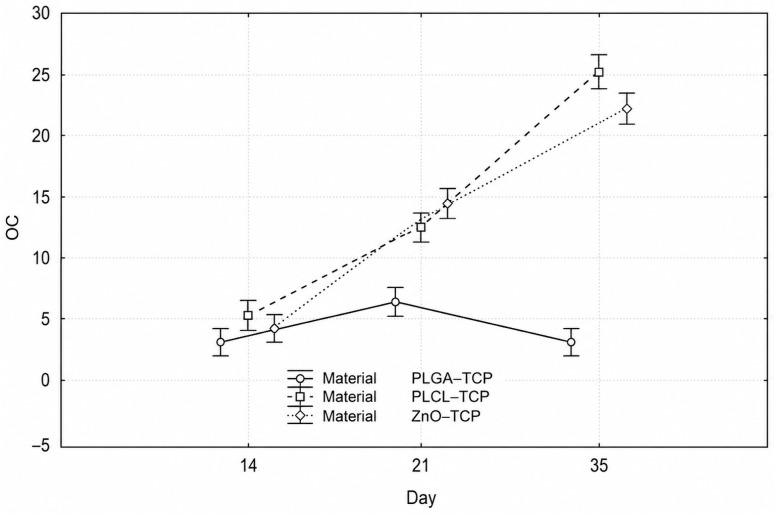
Osteogenesis-associated production of osteocalcin (OC) concentration by CMSCs cultured on β-TCP-containing composite biomaterials under non-osteogenic conditions (concentration (ng/mL).

The production of ALP, COL I, and OC by CMSCs cultured on β-TCP-containing composite biomaterials was evaluated during the 35-day cultivation period (Figure 4, Figure 5 and Figure 6).

Differences in COL I production (Figure 5) were prominent among the investigated biomaterials. At day 14, COL I levels were highest in the PLCL–TCP group (80.2 ± 1.0), followed by PLGA–TCP (75.1 ± 0.8) and ZnO–TCP (70.1 ± 1.1). After 21 days, PLCL–TCP exhibited a marked increase, reaching a maximum expression of 95.1 ± 1.2, while ZnO–TCP showed moderate levels (75.1 ± 1.2) and PLGA–TCP decreased to 65.1 ± 1.0. By day 35, PLCL–TCP maintained elevated production (85.1 ± 1.2), whereas ZnO–TCP decreased to 60.1 ± 1.2 and PLGA–TCP demonstrated the lowest expression (30.1 ± 0.8). Statistical analysis confirmed significant effects of material type, cultivation time, and their interaction (*p* < 0.001).

The production of OC (Figure 6) increased significantly during cultivation in the PLCL–TCP and ZnO–TCP groups, while PLGA–TCP exhibited consistently low production. At day 14, the highest OC production was observed in the PLCL–TCP group (5.1 ± 0.4), followed by ZnO–TCP (4.1 ± 0.4) and PLGA–TCP (3.1 ± 0.3). After 21 days, OC levels increased, with ZnO–TCP exhibiting 14.1 ± 1.1 and PLCL–TCP reaching 12.1 ± 1.0. By day 35, PLCL–TCP demonstrated the highest osteocalcin production (25.1 ± 1.3), and ZnO–TCP reached 22.1 ± 1.2, whereas PLGA–TCP remained at low levels (3.1 ± 0.3). Two-way ANOVA demonstrated significant effects of biomaterial type, cultivation time, and their interaction (*p* < 0.001). Notably, while most material and time differences were highly significant, the Scheffé post hoc test revealed no statistically significant difference in overall OC production between the PLCL–TCP and ZnO–TCP material groups (*p* = 0.2497).

### 3.4. Morphological and Chemical Characterization (SEM and EDX)

The morphological and chemical changes in the scaffold surfaces following 35 days of CMSC cultivation were evaluated using SEM and EDX analysis. While all investigated composites maintained their macroscopic structural integrity throughout the extended cultivation period, distinct material-specific modifications in surface topography, porosity, and elemental distribution were observed at the microscopic level.

#### 3.4.1. PLCL–TCP (Poly(ε-caprolactone-co-lactide) Containing β-Tricalcium phosphate)

**Figure 7:**
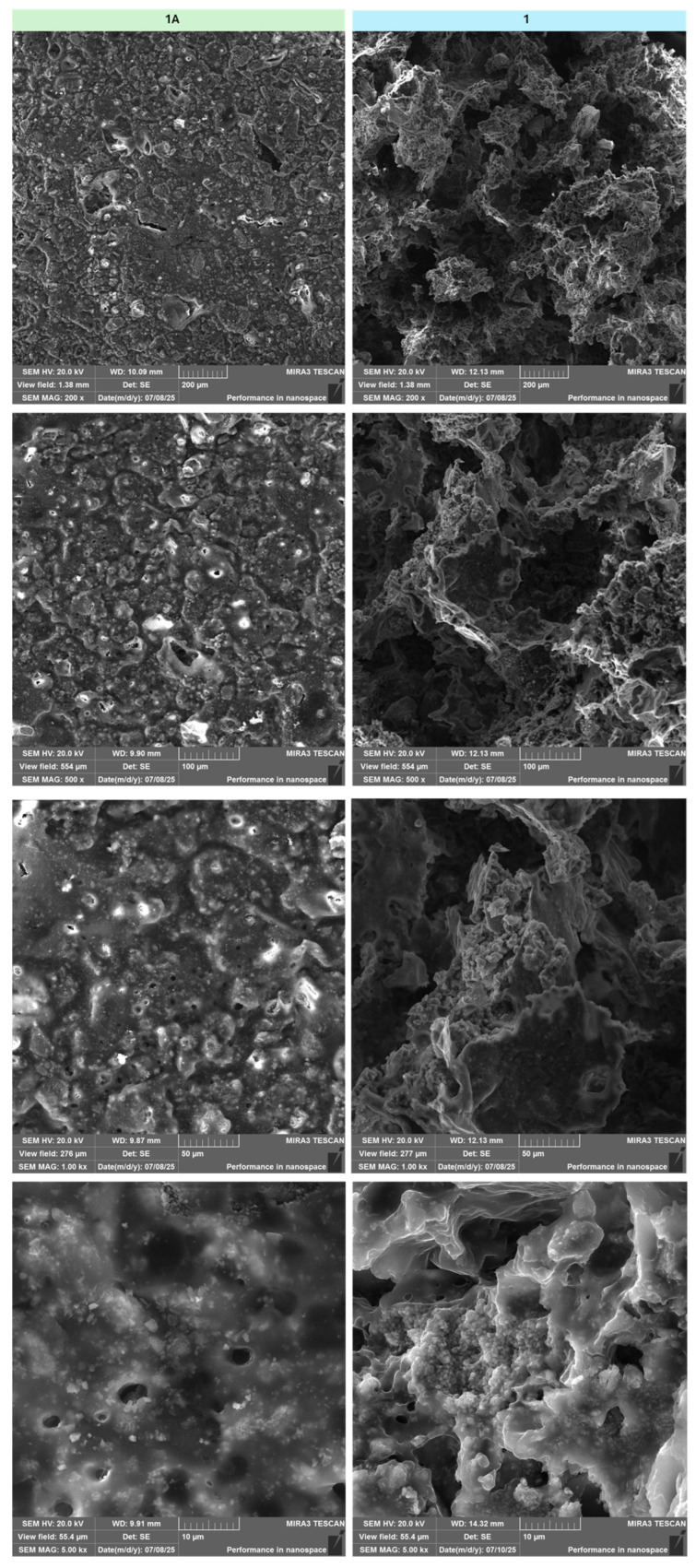
SEM micrographs of PLCL–TCP scaffolds before and after cultivation with chorion-derived mesenchymal stem cells (CMSCs). The scaffold prior to cultivation (**1A**) exhibited a compact and relatively homogeneous surface morphology with moderate roughness. Following cultivation (**1**), the scaffold developed a more heterogeneous and porous architecture characterized by interconnected surface features, increased roughness, and fibrillar-like structures. Representative micrographs are shown at magnifications of 200×, 500×, 1.00 k×, and 5.00 k×.

SEM analysis revealed noticeable differences in the surface morphology of PLCL–TCP before and after CMSC cultivation (Figure 7). The scaffold prior to cultivation exhibited a relatively compact and homogeneous surface with moderate roughness and discrete particulate features. Following cultivation, the scaffold surface appeared more heterogeneous and porous, displaying a more developed three-dimensional architecture. Numerous interconnected cavities, irregular surface structures, and fibrillar-like features were observed throughout the scaffold surface. Despite these morphological changes, the overall scaffold architecture remained preserved, indicating good structural stability during long-term cultivation. The observed differences may reflect changes associated with cultivation conditions and exposure to the biological environment.

**Figure 8:**
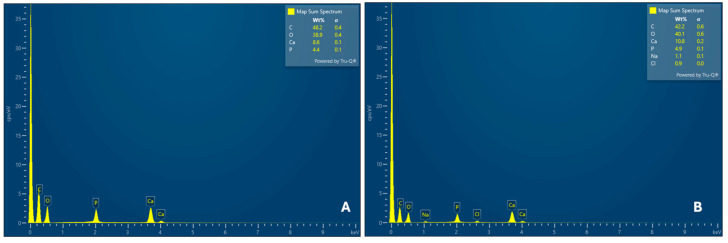
Representative EDX map-sum spectra of PLCL–TCP scaffolds before and after cultivation with chorion-derived mesenchymal stem cells (CMSCs). (**A**) Untreated scaffold prior to cultivation. (**B**) Scaffold after 35 days of cultivation. The x-axis represents X-ray energy (keV) and the y-axis indicates signal intensity (cps/eV).

Comparison of EDX spectra obtained before and after cultivation demonstrated preservation of the characteristic polymer–ceramic composition of PLCL–TCP (Figure 8). In both samples, carbon and oxygen represented the predominant elements, while calcium and phosphorus peaks confirmed the continued presence of the β-TCP phase. Following cultivation, the relative abundance of calcium increased from 8.6 wt.% to 10.8 wt.% and phosphorus from 4.4 wt.% to 4.9 wt.%. In contrast, carbon decreased from 48.2 wt.% to 42.2 wt.%. Minor sodium (1.1 wt.%) and chlorine (0.9 wt.%) signals were additionally detected after cultivation. These findings indicate modifications of the surface chemical environment during biological exposure while maintaining the characteristic composition of the PLCL–TCP composite.

**Figure 9:**
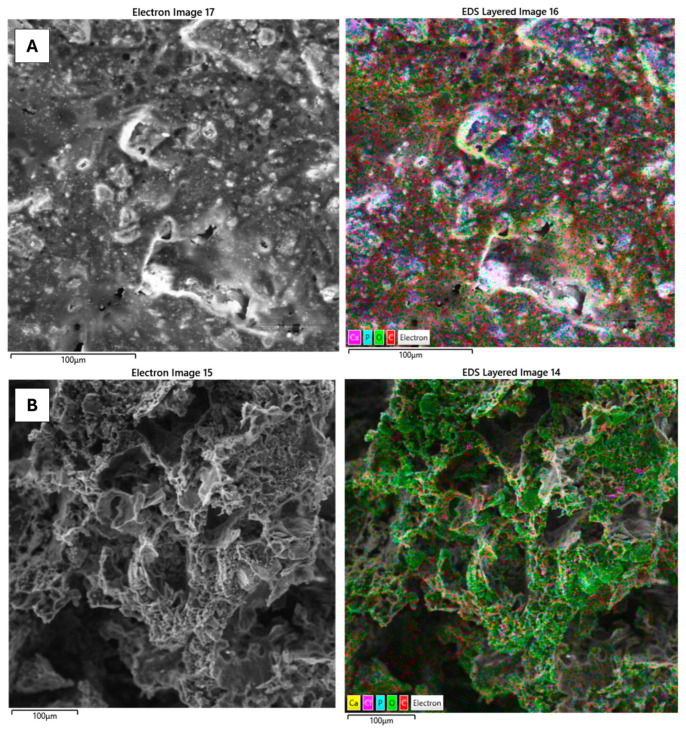
SEM micrographs and corresponding EDX elemental overlay maps of PLCL–TCP scaffolds. (**A**) Scaffold prior to cultivation exhibiting a relatively compact surface morphology. (**B**) Scaffold after 35 days of cultivation demonstrating a more heterogeneous, porous architecture. Scale bars = 100 μm.

EDX elemental mapping confirmed the preservation of the characteristic surface composition of PLCL–TCP following CMSC cultivation (Figure 9). Prior to cultivation, carbon, oxygen, calcium, and phosphorus were distributed relatively homogeneously across the scaffold surface. After 35 days, these principal elements remained present, with calcium and phosphorus continuing to exhibit a spatially associated distribution characteristic of calcium phosphate-rich regions.

**Figure 10:**
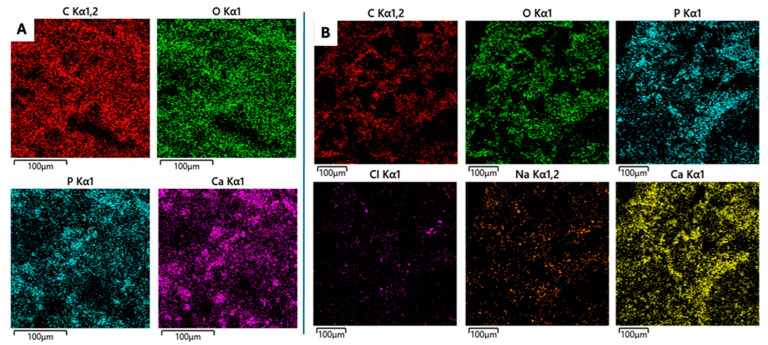
EDX elemental maps of PLCL–TCP scaffolds before and after cultivation with chorion-derived mesenchymal stem cells (CMSCs). (**A**) Untreated scaffold prior to cultivation showing the distribution of carbon (C), oxygen (O), phosphorus (P), and calcium (Ca). (**B**) Scaffold after 35 days of cultivation showing the distribution of carbon (C), oxygen (O), phosphorus (P), calcium (Ca), chlorine (Cl), and sodium (Na). Scale bars = 100 μm.

Further detailed elemental mapping (Figure 10) corroborated these findings. While minor sodium and chlorine signals were newly detected following cultivation—likely deposited from the culture environment—no major alterations in the overall elemental distribution were observed. This indicates that the scaffold successfully maintained its stable polymer–ceramic composition during long-term biological exposure.

#### 3.4.2. PLGA–TCP (Poly(lactide-co-glycolide) Containing β-Tricalcium phosphate)

**Figure 11:**
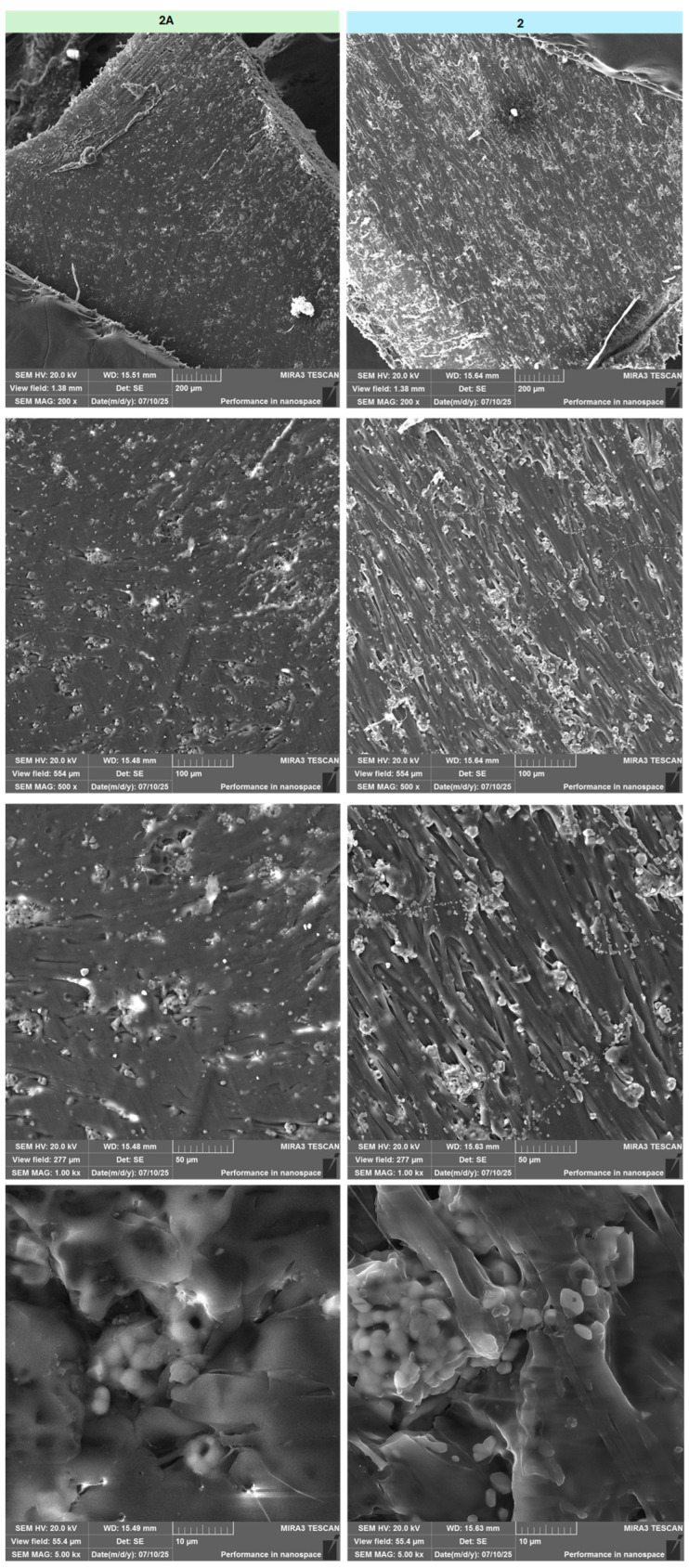
SEM micrographs of PLGA–TCP scaffolds before and after cultivation with chorion-derived mesenchymal stem cells (CMSCs). Untreated scaffold prior to cultivation (**2A**). Scaffold following 35 days of cultivation (**2**). Representative micrographs are shown at increasing magnifications from left to right: 200×, 500×, 1.00 k×, and 5.00 k×.

SEM analysis demonstrated noticeable morphological changes on the surface of PLGA–TCP following CMSC cultivation (Figure 11). Prior to cultivation, the scaffold exhibited a relatively compact and homogeneous surface morphology characterized by longitudinal structural features and moderate surface roughness. After cultivation, the overall scaffold architecture remained preserved; however, the surface became more heterogeneous and displayed an increased number of particulate deposits and irregular surface features. Higher magnification images revealed localized accumulations of granular structures and partial masking of the original surface topography. These observations indicate substantial interaction between the scaffold surface and the biological environment during long-term cultivation.

**Figure 12:**
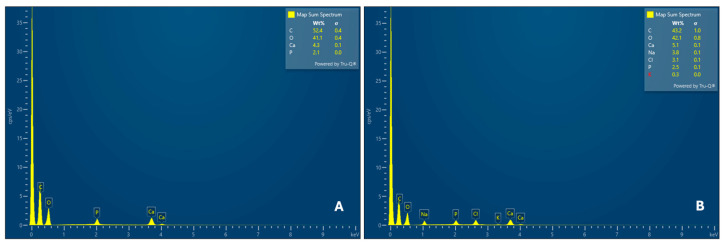
Representative EDX map-sum spectra of PLGA–TCP scaffolds before and after cultivation with chorion-derived mesenchymal stem cells (CMSCs). (**A**) Untreated scaffold prior to cultivation. (**B**) Scaffold after 35 days of cultivation. The x-axis represents X-ray energy (keV) and the y-axis indicates signal intensity (cps/eV).

EDX analysis of PLGA–TCP revealed moderate changes in surface elemental composition following CMSC cultivation (Figure 12). Prior to cultivation, the scaffold surface was dominated by carbon (52.4 wt.%) and oxygen (41.1 wt.%) originating from the poly(lactide-co-glycolide) matrix, while calcium (4.3 wt.%) and phosphorus (2.1 wt.%) confirmed the presence of the β-TCP phase. Following cultivation, the relative abundance of calcium and phosphorus increased slightly to 5.1 wt.% and 2.5 wt.%, respectively, whereas carbon decreased to 43.2 wt.%. In addition, sodium (3.8 wt.%), chlorine (3.1 wt.%), and trace potassium (0.3 wt.%) were detected after cultivation. These findings indicate modifications of the surface chemical environment during biological exposure and suggest increased interaction of the scaffold surface with the culture medium.

**Figure 13:**
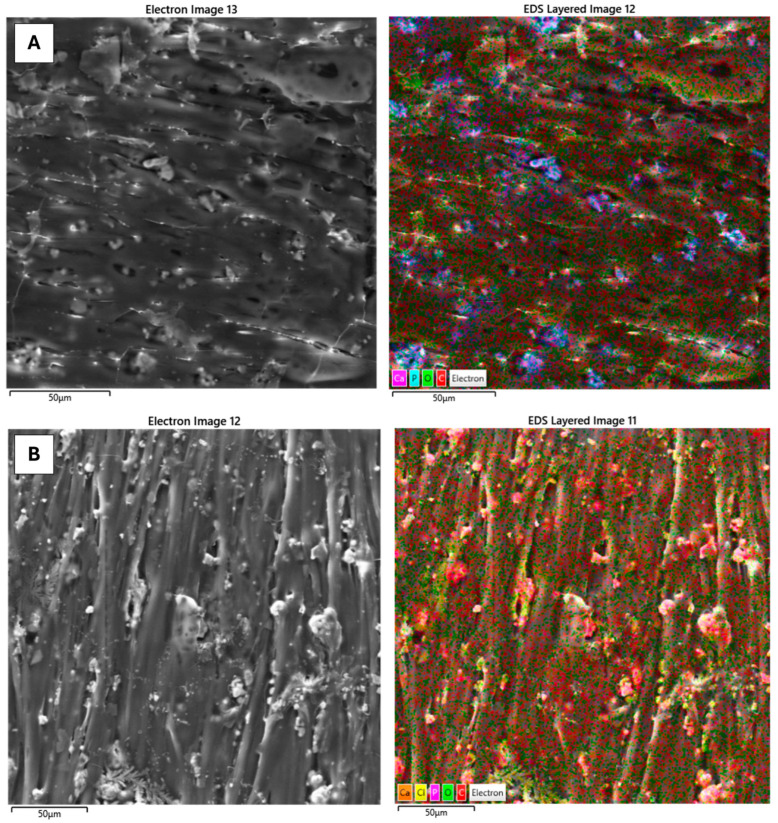
SEM micrographs and corresponding EDX elemental overlay maps of PLGA–TCP scaffolds before and after cultivation with chorion-derived mesenchymal stem cells (CMSCs). (**A**) Scaffold prior to cultivation showing the baseline surface architecture and corresponding distribution of carbon (C), oxygen (O), phosphorus (P), and calcium (Ca). (**B**) Scaffold after 35 days of cultivation showing surface topography alongside mapped elements, including the detection of trace chlorine (Cl). Scale bar = 50 μm.

SEM imaging combined with EDX elemental mapping demonstrated preservation of the characteristic surface architecture of PLGA–TCP following CMSC cultivation (Figure 13). Prior to cultivation, the scaffold exhibited a relatively compact surface with visible longitudinal structural features and a homogeneous distribution of carbon, oxygen, calcium, and phosphorus. Following cultivation, the overall surface topography remained preserved; however, increased surface heterogeneity and the presence of numerous particulate deposits were observed. Elemental mapping confirmed the continued presence of calcium and phosphorus throughout the scaffold surface after cultivation. The distribution of these elements remained associated with calcium phosphate-rich regions, indicating retention of the β-TCP component during long-term biological exposure.

**Figure 14:**
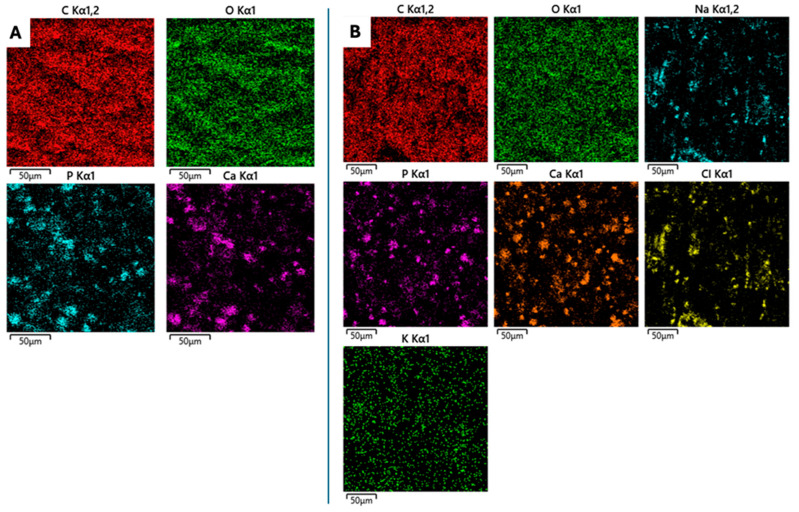
EDX elemental maps of PLGA–TCP scaffolds before and after cultivation with chorion-derived mesenchymal stem cells (CMSCs). (**A**) Untreated scaffold prior to cultivation showing the distribution of carbon (C), oxygen (O), phosphorus (P), and calcium (Ca). (**B**) Scaffold after 35 days of cultivation showing the distribution of carbon (C), oxygen (O), phosphorus (P), calcium (Ca), sodium (Na), chlorine (Cl), and potassium (K). Scale bar = 50 μm.

EDX elemental mapping confirmed preservation of the characteristic elemental composition of PLGA–TCP following CMSC cultivation (Figure 14). Prior to cultivation, carbon, oxygen, calcium, and phosphorus were distributed throughout the scaffold surface, with calcium and phosphorus exhibiting spatial overlap corresponding to β-TCP-rich regions. Following cultivation, the same principal elements remained present and maintained a comparable distribution pattern. In addition, sodium, chlorine, and trace potassium were detected after cultivation. These elements were dispersed across the scaffold surface without forming distinct localized accumulations. The continued co-localization of calcium and phosphorus indicates preservation of the β-TCP phase during long-term biological exposure.

#### 3.4.3. ZnO–TCP (Zinc Oxide Containing β-Tricalcium phosphate)

**Figure 15:**
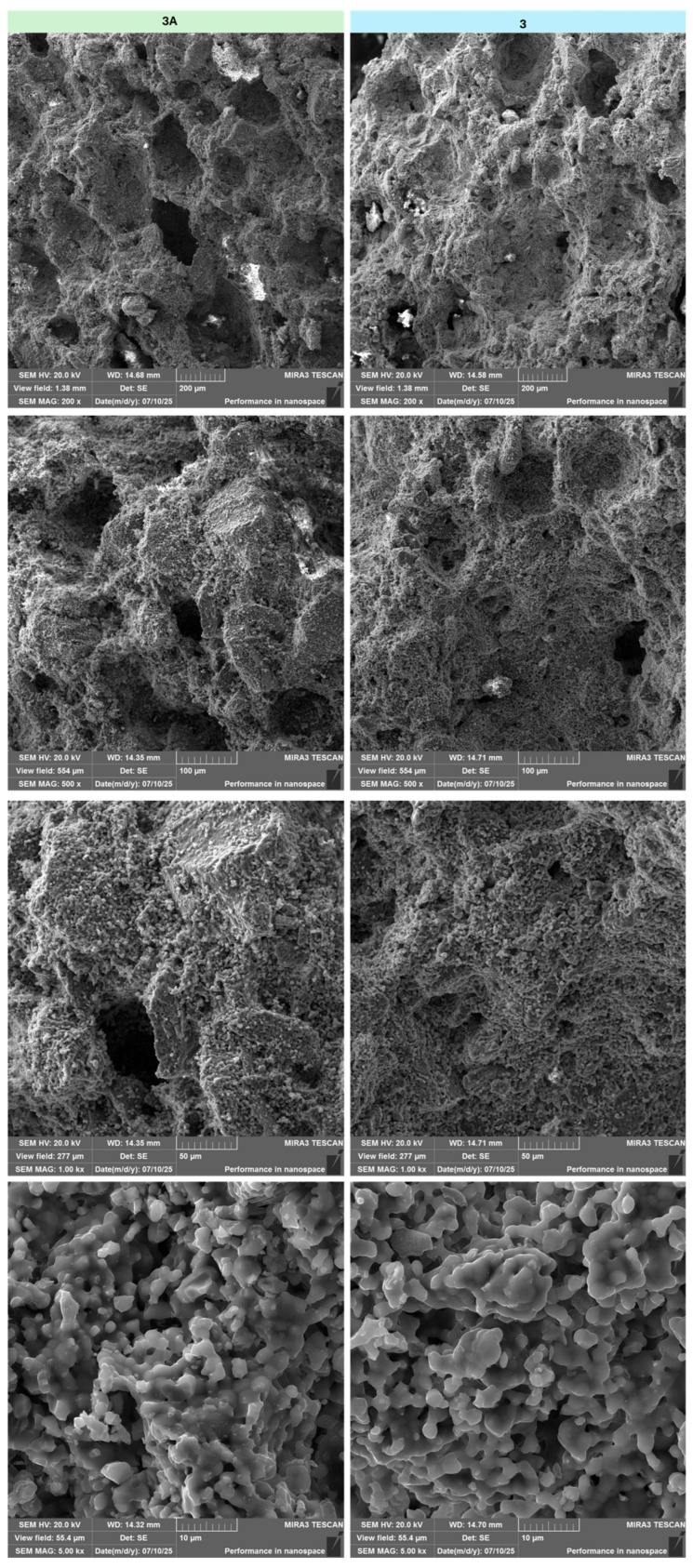
SEM micrographs of ZnO–TCP scaffolds before and after cultivation with chorion-derived mesenchymal stem cells (CMSCs). Untreated scaffold prior to cultivation (**3A**). Scaffold following 35 days of cultivation (**3**). Representative micrographs are shown at increasing magnifications from left to right: 200×, 500×, 1.00 k×, and 5.00 k×.

SEM analysis of ZnO–TCP revealed a highly porous and heterogeneous surface morphology both before and after CMSC cultivation (Figure 15). Prior to cultivation, the scaffold exhibited a rough surface composed of densely packed granular structures and interconnected pores. Higher magnification images demonstrated a characteristic particulate morphology consistent with the inorganic nature of the composite. Following cultivation, the overall surface architecture remained largely unchanged. The scaffold retained its porous structure and granular morphology, while only minor surface modifications were observed. Compared with PLCL–TCP and PLGA–TCP, the morphological changes associated with cultivation appeared less pronounced, indicating good structural stability of the ZnO–TCP scaffold during long-term biological exposure.

**Figure 16:**
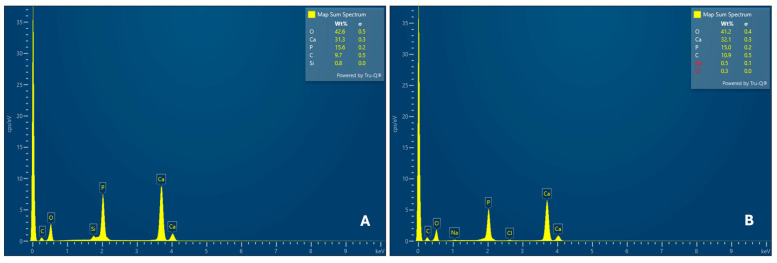
Representative EDX map-sum spectra of ZnO–TCP scaffolds before and after cultivation with chorion-derived mesenchymal stem cells (CMSCs). (**A**) Untreated scaffold prior to cultivation. (**B**) Scaffold after 35 days of cultivation. The x-axis represents X-ray energy (keV) and the y-axis indicates signal intensity (cps/eV).

EDX analysis of ZnO–TCP revealed only minor changes in surface elemental composition following CMSC cultivation (Figure 16). Prior to cultivation, the scaffold surface was dominated by oxygen (42.6 wt.%), calcium (31.3 wt.%), and phosphorus (15.6 wt.%), confirming the highly inorganic character of the composite. Following cultivation, the relative abundances of the principal elements remained largely unchanged, with calcium and phosphorus reaching 32.1 wt.% and 15.0 wt.%, respectively. Small amounts of sodium (0.5 wt.%) and chlorine (0.3 wt.%) were detected after cultivation, while the overall elemental profile remained stable. These findings indicate preservation of the characteristic calcium phosphate-rich surface composition during long-term biological exposure.

**Figure 17:**
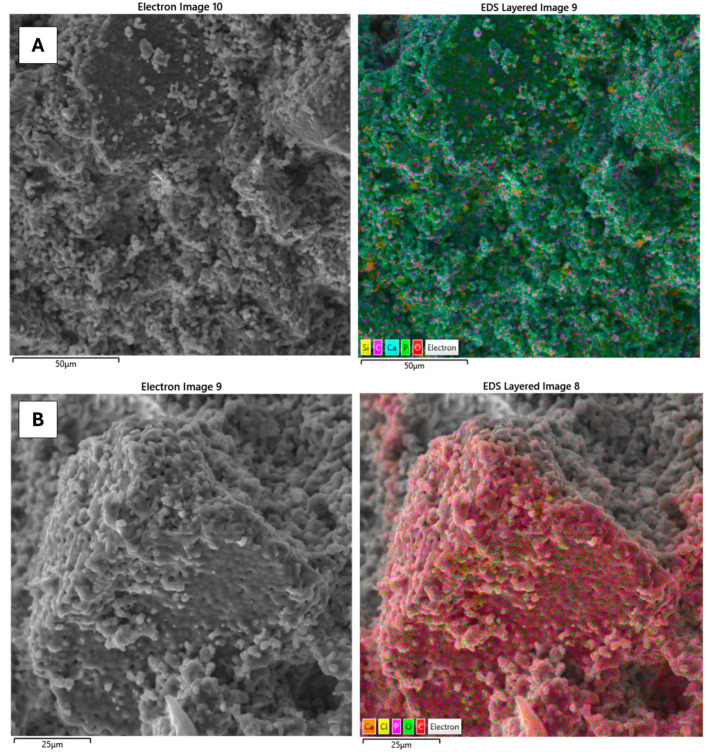
SEM micrographs and corresponding EDX elemental overlay maps of ZnO–TCP scaffolds before and after cultivation with chorion-derived mesenchymal stem cells (CMSCs). (**A**) Scaffold prior to cultivation showing the baseline surface architecture and corresponding distribution of silicon (Si), carbon (C), calcium (Ca), phosphorus (P), and oxygen (O). (**B**) Scaffold after 35 days of cultivation showing surface topography alongside mapped elements, including calcium (Ca), chlorine (Cl), phosphorus (P), oxygen (O), and carbon (C). Scale bars = 50 μm (**A**) and 25 μm (**B**).

SEM imaging combined with EDX elemental mapping demonstrated preservation of the characteristic surface organization of ZnO–TCP following CMSC cultivation (Figure 17). Prior to cultivation, the scaffold exhibited a densely packed granular morphology accompanied by a homogeneous distribution of calcium and phosphorus throughout the analyzed surface. Following cultivation, the overall morphology remained largely unchanged, and the characteristic granular architecture was retained. Elemental mapping confirmed continued spatial overlap of calcium and phosphorus, indicating preservation of calcium phosphate-rich regions after biological exposure. No major alterations in elemental distribution were observed, supporting the high structural and chemical stability of the ZnO–TCP scaffold during long-term cultivation.

**Figure 18:**
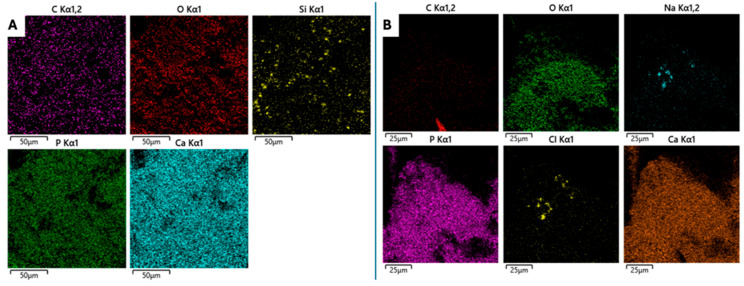
EDX elemental maps of ZnO–TCP scaffolds before and after cultivation with chorion-derived mesenchymal stem cells (CMSCs). (**A**) Untreated scaffold prior to cultivation showing the distribution of carbon (C), oxygen (O), silicon (Si), phosphorus (P), and calcium (Ca). (**B**) Scaffold after 35 days of cultivation showing the distribution of carbon (C), oxygen (O), sodium (Na), phosphorus (P), chlorine (Cl), and calcium (Ca). Scale bars = 50 μm (**A**) and 25 μm (**B**).

EDX elemental mapping confirmed the preservation of the characteristic elemental organization of ZnO–TCP following CMSC cultivation (Figure 18). Prior to cultivation, calcium and phosphorus were homogeneously distributed throughout the analyzed surface and exhibited substantial spatial overlap, consistent with the calcium phosphate-rich nature of the composite. Oxygen showed a similarly uniform distribution, while carbon and trace silicon were present at lower levels. Following cultivation, calcium and phosphorus remained the dominant elements and retained a comparable distribution pattern. Minor amounts of sodium and chlorine were detected after cultivation; however, these elements appeared only as dispersed signals and did not alter the overall elemental organization of the scaffold surface. The results indicate maintenance of the characteristic calcium phosphate-rich composition during long-term biological exposure.

## 4. Discussion

The present study investigated the biological response of chorion-derived mesenchymal stem cells (CMSCs) cultured on β-TCP-containing composite scaffolds under non-osteogenic conditions. Unlike many studies employing osteogenic supplements such as dexamethasone, β-glycerophosphate, and ascorbic acid, the current experimental design aimed to evaluate the intrinsic ability of scaffold properties to influence cell behavior. The results demonstrated that all investigated materials supported long-term CMSC viability and induced varying levels of osteogenesis-associated marker production despite the absence of chemical osteogenic stimulation.

All investigated scaffolds supported long-term CMSC viability, as confirmed by MTT analysis. The sustained metabolic activity observed throughout the 35-day cultivation period indicates good cytocompatibility of all β-TCP-containing composites. These findings are consistent with previous reports demonstrating favorable interactions between mesenchymal stem cells and calcium phosphate-based biomaterials. Calcium phosphate-containing scaffolds have repeatedly been shown to provide a suitable environment for stem cell adhesion, proliferation, and osteogenic commitment due to their chemical similarity to the mineral phase of native bone [33]. Müller et al. demonstrated that calcium phosphate surfaces can promote osteogenic differentiation of mesenchymal stem cells even in the absence of osteogenic supplements, highlighting the intrinsic osteoinductive potential of these materials [10].

Despite comparable cell viability, significant differences were observed in osteogenesis-associated marker production. PLCL–TCP consistently exhibited the highest ALP activity and COL I production, whereas PLGA–TCP showed the weakest response. Since ALP represents an early marker of osteogenic differentiation [34] and COL I reflects extracellular matrix synthesis [35], these findings suggest that PLCL–TCP provided the most favorable microenvironment for osteogenic commitment and matrix formation. Interestingly, ALP values gradually decreased over time while OC concentrations increased, particularly in PLCL–TCP and ZnO–TCP. This temporal pattern is consistent with the normal progression of osteogenic differentiation, during which early markers decline as cells transition toward a more mature osteoblastic phenotype characterized by increased osteocalcin production [36]. Similar trends have been reported in previous studies of mesenchymal stem cell osteogenesis, where ALP expression peaks during the early stages of differentiation and subsequently decreases as late-stage markers such as osteocalcin become upregulated. Birmingham et al. demonstrated that ALP activity reaches maximal levels during matrix maturation and declines during the mineralization phase, coinciding with increased osteocalcin expression and osteoblast maturation [37].

The ability of the investigated scaffolds to stimulate osteogenesis-associated responses without biochemical induction is consistent with current understanding of calcium phosphate-mediated osteogenesis [38]. Shih et al. demonstrated that calcium phosphate-bearing matrices directly regulate stem cell fate through material-driven signaling mechanisms, emphasizing that scaffold composition itself can influence osteogenic differentiation [11]. Similarly, Hatt et al. reported that β-TCP-containing scaffolds may serve as an effective source of phosphate ions capable of supporting osteogenic differentiation of mesenchymal stromal cells [39]. Together, these findings support the interpretation that β-TCP contributed substantially to the osteogenic responses observed in the present study. Importantly, these responses were observed in the absence of osteogenic supplements, suggesting that scaffold-derived physicochemical cues contributed substantially to the regulation of CMSC behavior throughout the cultivation period.

Surface characterization further revealed distinct differences among the investigated composites. SEM analysis demonstrated pronounced surface remodeling of PLCL–TCP following cultivation, accompanied by an increase in detectable calcium and phosphorus content. In contrast, PLGA–TCP exhibited increased surface heterogeneity and the strongest accumulation of sodium, chlorine, and potassium ions derived from the culture medium. Although PLGA–TCP interacted with the surrounding environment, this behavior was not associated with enhanced osteogenic marker production. These observations suggest that simple adsorption of medium-derived ions is insufficient to explain osteogenic stimulation.

ZnO–TCP displayed the highest detectable calcium and phosphorus content among all investigated materials both before and after cultivation. Surprisingly, however, ZnO–TCP did not induce the strongest osteogenic response. While OC production increased substantially during cultivation, ALP and COL I levels remained below those observed for PLCL–TCP. This finding suggests that the biological performance of composite scaffolds cannot be explained solely by the quantity of exposed calcium phosphate. Instead, the overall cellular response likely reflects a complex interplay between surface chemistry, scaffold architecture, porosity, topography, and ion release behavior. A similar observation was reported by Xiao et al. The authors emphasized that osteogenic outcomes on calcium phosphate-based biomaterials are strongly influenced by surface structure and microarchitecture in addition to chemical composition. Their review highlighted that variations in surface morphology, roughness, porosity, crystal size, and surface energy can significantly affect cell adhesion, proliferation, and differentiation, even among materials with comparable calcium phosphate content [12]. These findings are consistent with our results, where the scaffold exhibiting the highest calcium and phosphorus exposure did not necessarily produce the strongest osteogenic response.

Although all scaffolds maintained acceptable cell viability throughout the cultivation period, a gradual decline in metabolic activity was observed for PLGA–TCP at later time points. This phenomenon may be associated with the well-documented degradation behavior of PLGA. According to previous studies, the hydrolytic degradation of PLGA generates lactic and glycolic acids, which have been reported to contribute to local acidification within the scaffold microenvironment under certain conditions [40]. Such pH reductions have been reported to negatively affect cell viability, proliferation, and osteogenic differentiation, particularly during long-term culture [41]. Lu et al. highlighted the potential cytotoxic effects of acidic degradation products generated by aliphatic polyesters [42]. Similarly, Makadia et al. described how PLGA degradation results in acidic byproducts that may alter the local microenvironment and influence cellular responses [43]. Additional evidence was provided by Sung et al., where acidic degradation products were identified as an important factor affecting cell behavior within biodegradable polymer scaffolds [44]. Therefore, the reduced viability observed for PLGA–TCP in the present study may reflect not only differences in surface properties but also the biological consequences of progressive polymer degradation.

Collectively, the present findings indicate that all investigated β-TCP-containing composites were capable of supporting CMSC survival and inducing measurable osteogenesis-associated responses under non-osteogenic conditions. Among the tested materials, PLCL–TCP demonstrated the most favorable overall biological profile, combining sustained metabolic activity with elevated ALP, COL I, and OC production. In contrast, PLGA–TCP exhibited lower osteogenic activity despite pronounced interaction with the culture environment, whereas ZnO–TCP demonstrated excellent structural and chemical stability but only moderate biological stimulation. These observations suggest that optimization of scaffold performance requires balancing calcium phosphate bioactivity with scaffold architecture and surface properties rather than maximizing calcium phosphate exposure alone.

## 5. Conclusions

This study evaluated the biological response of chorion-derived mesenchymal stem cells cultured on β-TCP -containing composite scaffolds under non-osteogenic conditions. All investigated materials supported long-term cell viability, confirming their cytocompatibility and suitability for extended in vitro cultivation. Despite the absence of osteogenic supplements, all β-TCP-containing composites induced measurable osteogenesis-associated responses, as evidenced by the production of ALP, COL I, and OC. However, significant differences were observed among the investigated materials. PLCL–TCP demonstrated the most favorable overall biological performance, characterized by sustained metabolic activity, elevated ALP and COL I production, and increased osteocalcin expression at later cultivation stages. PLGA–TCP supported CMSC viability but exhibited the weakest osteogenic response despite pronounced interaction with the culture environment. ZnO–TCP displayed the highest calcium phosphate surface contribution and structural and chemical stability throughout cultivation, although its osteogenic stimulation remained moderate compared with PLCL–TCP. SEM and EDX analyses revealed material-specific differences in surface morphology and elemental composition following cultivation. While all scaffolds preserved their overall structural integrity, PLCL–TCP showed the most pronounced surface changes, PLGA–TCP exhibited increased adsorption of medium-derived ions, and ZnO–TCP maintained a largely unchanged surface architecture and elemental distribution. These findings indicate that osteogenic responses cannot be explained solely by calcium phosphate content and are likely governed by a complex interplay of surface chemistry, scaffold architecture, and cell–material interactions. Overall, the results demonstrate that β-TCP-containing composite scaffolds are capable of modulating CMSC behavior under non-osteogenic conditions. Among the investigated materials, PLCL–TCP exhibited the most promising combination of biological activity and structural stability, suggesting its potential suitability for future bone tissue engineering applications.

## Figures and Tables

**Table 1:** Composition of culture medium.

Component		Content	Manufacturer
DMEM	Dulbecco’s Modified Eagle Medium	1:1	Gibco, Thermo Fisher Scientific, Carlsbad, CA, USA
α-MEM	α-Minimum Essential Medium	1:1	
FBS	Fetal bovine serum	10% (*v*/*v*)	
Antibiotic-Antimycotic	Penicillin–streptomycin–amphotericin B	1% (*v*/*v*)	

**Table 2:** Immunophenotypic characterization of CMSCs after the first passage.

Marker	Marker Type	Expression (%)	Interpretation
CD73	Mesenchymal	91.9	Strongly positive
CD29	Mesenchymal	88.3	Positive
CD44	Mesenchymal	77.6	Positive
CD14/CD19/CD34/CD45	Hematopoietic	2.9	Negative

## Data Availability

The original contributions presented in this study are included in the article. Further inquiries can be directed to the corresponding author.
